# Response to the Letter: Mean Platelet Volume and Related Parameters May Not Contribute to the Diagnosis in Patients with Ascending Thoracic Aortic Aneurysm

**DOI:** 10.21470/1678-9741-2021-0024

**Published:** 2022

**Authors:** Yusuf Kenan Tekin, Gülaçan Tekin, İlhan Korkmaz, Sefa Yurtbay

**Affiliations:** 1 Department of Emergency Medicine, Sivas Cumhuriyet University, Sivas, Turkey. E-mail: yktekin@hotmail.com; 2 Department of Cardiology, Faculty of Medicine, Sivas Cumhuriyet University, Sivas, Turkey.

**Dear Editor,**

We carefully read the comments of dear colleagues Cengiz Beyan and Esin Beyan^[1]^. about our article titled “Mean Platelet Volume and Related Parameters May not Contribute to the Diagnosis in Patients with Ascending Thoracic Aortic Aneurysm”^[2]^.

Beyan et al.^[1]^ commented that “The control group did not consist of healthy volunteers, and it was made up of individuals who applied in the hospital at the same time. The fact that the control group is not composed of healthy volunteers and does not represent the society makes it difficult to interpret the results obtained.”. Our study results have a statistically significant value, and while we have excluded the patients previously diagnosed with hematological malignancy, chronic obstructive pulmonary disease (or COPD), autoimmune liver disease, cirrhosis, metastatic bone marrow infiltration, acute or chronic inflammatory disease — such as physical trauma, tonsillitis, asthma, rheumatoid arthritis, and active hepatitis —, and current or recent treatment (in the past three months) with oral or intravenous steroids or other medications that might cause pancytopenia from the control group according to their background, it is possible that all the exclusion criteria cannot be made in a retrospective study and that there can be pre-analytical and analytical errors, but this does not change the statistical difference.

Additionally, Beyan et al.^[1]^ tried to draw attention that mean platelet volume (MPV) is a complete blood count parameter whose measurement has not been standardized to date and, therefore, it has been reported to have no role in diagnosis and prognosis of acquired diseases according to a study, but there are many studies about the prognostic value of MPV in many clinical diseases. Vardon-Bounes et al.^[3]^ reported that MPV was an independent predictive factor of 90-day mortality. They suggested that continuous monitoring of MPV may be a useful parameter to stratify mortality risk in septic shock. Ma et al.^[4]^ reported that high MPV can be considered as an independent biomarker for predicting three-month mortality in patients with hepatitis B virus-related decompensated cirrhosis. Lee et al.^[5]^ indicated that MPV measurements may be used as a prognostic marker of mortality in intensive care unit patients with pneumonia.

İt is a fact that MPV can increase after the blood has been in contact with ethylenediaminetetraacetic acid in the blood tube. Beyan et al.^[1]^ supported this fact with the study by Jackson SR et al.^[6]^. In that study, it was mentioned that “Following the collection of the blood to the blood tube, as soon as the platelets come into contact with ethylenediaminetetraacetic acid, which is used as an anticoagulant, their diameters increase up to 30% within the first two hours”. However, our emergency department is a three-step emergency service that provides 24-hour service, the blood tests requested from the patients are delivered to the laboratory within 2-3minutes and are processed. The time is not suitable for the enlargement of MPV.

For all the reasons explained above, we respectfully disagree with the opinions of our colleagues Cengiz Beyan and Esin Beyan.

## References

[1] Beyan C, Beyan E (2020). Mean platelet volume and related parameters may not contribute to the diagnosis in patients with ascending thoracic aortic aneurysm. Braz J Cardiovasc Surg.

[2] Tekin YK, Tekin G (2020). Mean platelet volume-to-platelet count ratio, mean platelet volume-to-lymphocyte ratio, and red blood cell distribution width-platelet count ratio as markers of inflammation in patients with ascending thoracic aortic aneurysm. Braz J Cardiovasc Surg.

[3] Vardon-Bounes F, Gratacap MP, Groyer S, Ruiz S, Georges B, Seguin T (2019). Kinetics of mean platelet volume predicts mortality in patients with septic shock. PLoS One.

[4] Ma Y, Quan W, Zhu H (2018). High mean platelet volume is associated with worse outcomes in patients with HBV-related decompensated cirrhosis. Ann Clin Lab Sci.

[5] Lee JH, Park M, Han S, Hwang JJ, Park SH, Park SY (2018). An increase in mean platelet volume during admission can predict the prognoses of patients with pneumonia in the intensive care unit: a retrospective study. PLoS One.

[6] Jackson SR, Carter JM (1993). Platelet volume: laboratory measurement and clinical application. Blood Rev.

